# Diet affordability: a key dimension in the assessment of sustainable food systems and healthy diets

**DOI:** 10.3389/fnut.2024.1399019

**Published:** 2024-08-12

**Authors:** Sylvia M. S. Chungchunlam, Paul J. Moughan

**Affiliations:** Riddet Institute, Massey University, Palmerston North, New Zealand

**Keywords:** diet cost, diet optimization model, linear programming (LP), nutrient adequacy, adult, protein, animal-source foods (ASF), plant-based food (PBF)

## Abstract

A promulgated global shift toward a plant-based diet is largely in response to a perceived negative environmental impact of animal food production, but the nutritional adequacy and economic implications of plant-sourced sustainable healthy dietary patterns need to be considered. This paper reviews recent modeling studies using Linear Programming to determine the respective roles of animal- and plant-sourced foods in developing a least-cost diet in the United States and New Zealand. In both economies, least-cost diets were found to include animal-based foods, such as milk, eggs, fish, and seafood, to meet the energy and nutrient requirements of healthy adults at the lowest retail cost. To model a solely plant-based least-cost diet, the prevailing costs of all animal-sourced foods had to be increased by 1.1 to 11.5 times their original retail prices. This led to the inclusion of fortified plant-based foods, such as fortified soymilk, and a plant-based diet that was considerably (34–45%) more costly. The first-limiting essential nutrients were mostly the vitamins and minerals, with special focus on pantothenic acid, zinc, and vitamin B-12, when transitioning from an animal- and plant-containing least-cost diet to a plant-only based least-cost diet. Modeled least-cost diets based on contemporary food costs include animal-sourced foods, at least for developed high-income US and NZ food economies, and potentially for developing low- and middle-income countries, such as Indonesia. Modeling of least-cost diets that consist exclusively of plant-based foods is feasible, but at a higher daily diet cost, and these diets are often close to limiting for several key nutrients. Diet affordability, as a key dimension of sustainable healthy diets, and the respective economic roles of animal- and plant-sourced foods need to be considered.

## 1 Introduction

Sustainable food systems and healthy diets should be considered around four interconnecting dimensions: environment, society and culture, nutrition, and affordability (1). While the environmental dimension is well studied (2, 3), the nutritional quality and particularly economic affordability of sustainable diets are often overlooked (4, 5). Recently, the perceived environmental impact of food production and consumption is underlying a move toward a planetary sustainable healthy diet that is mostly plant-based. This is largely argued for, based on the high-level comparison that animal-sourced foods give rise to more greenhouse gas emissions per kg of food than plant-sourced foods (6). However, the sustainable plant-forward EAT-Lancet diet has been found to be nutritionally inadequate for calcium, iron, zinc, and vitamin B-12, and unaffordable at a median global cost of US $ 2.84 per person per day (2011 food prices) for 24% of the world’s population (7, 8). Diet affordability needs to be taken into account when considering globally sustainable dietary patterns, as the monetary cost of foods is a crucial determinant of food choice, diet quality, and food and nutrient security (5, 8–11).

The modeling of cost-minimized diets that meet recommended energy and essential nutrient requirements and are most affordable, is routinely conducted (9–18). The global median cost of a nutritionally adequate least-cost diet was found to be US $ 1.35 per day for the year 2011 (14), and US $ 2.32 per day for the year 2017 (17). These diet costs were based on food retail prices converted from local currency into US dollars in terms of purchasing power parity (PPP). The modeled diet was considered nutritionally adequate when it met the energy (2,329 kcal) and nutritional requirements for half of the population of healthy non-pregnant, non-lactating, 30-year-old adult women, as defined as estimated average requirements (EARs) or harmonized average requirements (H-ARs) (14, 17). In comparison, when nutritional requirements were based on recommended dietary allowances (RDAs), to estimate intake levels adequate for 97–98% of a healthy population, or adequate intakes (AIs), the median daily diet cost for men and women aged 19–50 years, was found to be US $ 2.62 and 2.45 (US $ PPP for the year 2017), respectively (18). On the other hand, the average daily diet cost for an average adult aged 19–50 years was US $ 2.71 (18).

A common approach to evaluate diet affordability is to use Linear Programming (LP) as a mathematical dietary optimization tool to minimize dietary cost under a given set of linear constraints (19–21). Here, LP provides unique solutions for the mixtures of foods available in the market that meet all the nutritional requirements of the adult, but do so at the lowest price possible. The commonly applied LP does not necessarily give rise to practical dietary patterns, but rather highlights the role of key food groups in assisting to meet nutrient needs at the lowest cost. LP allows the interrogation of multiple food mixtures and identifies the one dietary combination that meets all the stated nutrient requirements of the adult at the lowest cost. The purpose of this paper is to review recent country-specific LP modeling studies to determine the inclusion levels of animal- and plant-sourced foods in the formulation of nutrient adequate dietary patterns at the lowest dietary cost. This paper brings together our previously reported LP modeling work in the United States (US) (22) and New Zealand (NZ) (23), and ongoing LP modeling research in developing countries. Moreover, diet cost is an important focal point of attention when transitioning from a diet that contains animal- and plant-sourced foods to a plant-only based vegan diet. The extent to which the relative prices of animal-sourced foods needed to be increased to be excluded from nutrient adequate least-cost dietary patterns and the economic feasibility of plant-only nutrient adequate least-cost dietary patterns were evaluated (22, 23).

## 2 Modeling of least-cost dietary patterns

Dietary optimization using the LP approach involves the minimization or maximization of a linear function of a set of decision variables, while subjected to several linear constraints (19–21). LP can take into account simultaneously food costs, the supply of locally consumed foods, food serving sizes, food nutritional compositional data, and energy and nutritional intake requirements, in the formulation of least-cost (most affordable), nutrient adequate, and culturally acceptable dietary patterns. Here, the LP model aimed to minimize the cost of the optimal dietary solution by changing the decision variables, which were the quantities and corresponding costs of selected foods, according to the following equation:


$$f\left(x\right)=\overset{N_{f}}{\underset{i=1}{\sum }}c_{i}x_{i}$$

where $f\left(x\right)$ is the diet cost, $N_{f}$ is the number of foods included in the LP analysis, $c_{i}$ is the cost per unit quantity of food *i*, and $x_{i}$ is the unit quantity of food *i*. The linear constraints applied in the LP model were daily estimated energy requirement, daily minimum and upper intake limits of nutrient requirements, and maximum limits on daily food serving sizes, and can be expressed using the following Equations 1–3, respectively.


(1) $$\overset{N_{f}}{\underset{i=1}{\sum }}e_{i}x_{i}=E$$

(2) $$m_{j}\left(j=1,2,\ldots ,N_{n}\right)\leq \overset{N_{f}}{\underset{i=1}{\sum }}n_{ij}x_{i}\leq u_{j}\;\left(j=1,2,\ldots ,N_{n}\right)$$

(3) $$0\leq x_{i}\leq 3r_{i}\;\left(i=1,2,\ldots ,N_{f}\right)$$

where $N_{f}$ is the number of foods included in the LP analysis, $e_{i}$ is the energy value per unit quantity of food *i,* $x_{i}$ is the unit quantity of food *i*, E is the daily estimated energy requirement to meet, $m_{j}$ is the daily minimum required intake level of nutrient *j,* $N_{n}$ is the number of nutrients included in the LP analysis, $n_{ij}$ is the amount of nutrient *j* per unit quantity of food *i,* $u_{j}$ is the daily upper intake limit of nutrient *j,* and $r_{i}$ is the daily recommended serving size for food *i*.

At a country-specific level, the LP approach was used to model nutrient adequate least-cost diets for adults in the US (22), NZ (23), and developing countries, such as Indonesia. An empirical approach was used for the linear constraints on food serving sizes, based on the assumption that individuals commonly consume three main meals per day, to limit the daily maximum allowable amount of each food or food subgroup to be no more than three servings per day. In the US (22), some additional pragmatic constraints were applied, to limit energy-rich foods (bread and bread rolls, tortillas, and rice) to no more than 2 servings per day, and to limit fat-rich foods (margarine and vegetable spreads, peanut butter, mayonnaise and salad dressings) to no more than 1 serving per day. Moreover, in NZ (23), margarine was limited to no more than two servings per day, rather than 3 servings per day, for the least-cost modeled diet to be within the acceptable macronutrient distribution of 20–35% of energy from fat. As the modeling study in Indonesia is preliminary, each food or food subgroup was initially constrained to be selected to no more to one serving per day. The constraints for daily energy and nutrient requirements of average adults aged 19–50 years, that were applied in the LP modeling studies in the US (22), NZ (23), and Indonesia are given in Table 1. Several least-cost dietary scenarios were explored in a step-wise manner, to evaluate dietary LP model outcomes for nutritional adequacy and cost.

**Table 1:** The nutritional constraints applied in the linear programming modeling analyses of nutrient adequate least-cost dietary patterns in the United States (22), New Zealand (23), and Indonesia, as daily energy and minimum level of nutrients required by average adults aged 19–50 years.

	United States	New Zealand	Indonesia
Energy	2,600 kcal	2,665 kcal	2,400 kcal
Carbohydrate	130 g		
Dietary fiber	31.5 g	27.5 g	33.75 g
Linoleic acid	14.5 g	10.5 g	
α-linolenic acid	1.35 g	1.05 g	
Protein	50.8 g	55 g	62.5 g
Calcium	1,000 mg	1,000 mg	1,000 mg
Chromium		30 μg	
Copper	0.9 mg	1.45 mg	0.9 mg
Iron	13 mg	13 mg	13.5 mg
Magnesium	355 mg	362.5 mg	
Manganese	2.05 mg	5.25 mg	
Molybdenum		45 μg	
Phosphorus	700 mg	1,000 mg	700 mg
Potassium	4,700 mg	3,300 mg	4,700 mg
Selenium	55 μg	65 μg	
Sodium	1,500 mg	670 mg	1,500 mg
Zinc	9.5 mg	11 mg	9.5 mg
Biotin		27.5 μg	
Choline	487.5 mg		
Folate	400 μg (DFE)	400 μg (DFE)	
Niacin	15 mg	15 mg	15 mg
Pantothenic acid	5 mg	5 mg	
Riboflavin	1.2 mg	1.2 mg	1.2 mg
Thiamin	1.15 mg	1.15 mg	1.15 mg
Vitamin A	800 μg (RAE)	800 μg (RE)	625 μg (RE)
Vitamin B-6	1.3 mg	1.3 mg	
Vitamin B-12	2.4 μg	2.4 μg	
Vitamin C	82.5 mg	45 mg	82.5 mg
Vitamin D	15 μg	5 μg	
Vitamin E	15 mg	8.5 mg	
Vitamin K	105 μg	65 μg	

DFE, Dietary Folate Equivalent; RAE, Retinol Activity Equivalent; RE, Retinol Equivalent. Average adult daily nutrient requirements values were sourced from the Institute of Medicine (24), National Health and Medical Research Council (25), and Ministry of Health (26), for the United States, New Zealand, and Indonesia, respectively.

### 2.1 Least-cost diets in the United States

The modeled nutrient adequate baseline least-cost diet in the US, using the most up-to-date, reliable, and comprehensive data on foods and food prices, was shown to have a daily diet cost of US $ 1.98 (2009–2010 US food prices), and comprised dairy milk, eggs, and fish as animal-sourced foods among the 15 foods in the diet (22). Milk (26%), fortified breakfast cereals (14.2%), potatoes (12.6%), and legumes (12.4%) largely contributed to the total diet cost. The fat-rich foods, such as margarine (1.4%) and mayonnaise (1.5%), and the carbohydrate-rich foods, such as corn tortillas (1.4%) and bread rolls (2.3%), accounted the least to total diet cost.

Increases in the baseline national retail prices of all animal-sourced foods by 5, 10, 15 or 20% were found to marginally change dietary composition, and to gradually and slightly increase diet cost up to US $ 2.14 per day (22). To model a dietary scenario whereby all animal-derived foods were no longer included in the least-cost diets by incrementally (5%) increasing food prices, the prices of selected animal-sourced foods had to be increased by 2.0 to 11.5 times their baseline costs (Table 2). The resulting plant-only least-cost diet contained 14 foods and had a daily diet cost of US $ 3.61 (22). The greatest contributors to total diet cost were fortified soymilk (37%), legumes (13.3%), fortified breakfast cereals (12.7%), and cabbage (9.7%). Unsurprisingly, energy-dense foods, such as corn tortillas (0.8%), margarine (1.1%), and vegetable oils (2.5%), contributed the least to total diet cost.

**Table 2:** The extent by which prevailing prices of animal-sourced foods selected in the linear programming modeling analyses of least-cost dietary patterns needed to be increased for their exclusion, in the United States (US) and New Zealand (NZ).

	United States (US)	New Zealand (NZ)
Food group	2009–2010 food prices	2020 food prices
Milk	8.0x	2.20x
Eggs	11.5x	1.80x
Fish	6.5x	2.30x
Seafood		10.30x
Chicken	5.0x	1.95x
Turkey	3.0x	
Beef	5.5x	
Pork	2.5x	
Lamb		1.25x
Cold cuts and cured meats	2.0x	
Sausages		1.05x
Cheese	3.0x	3.95x
Yogurt	2.5x	
Ice cream	2.0x	
Mayonnaise (containing eggs)	5.0x	
Bread rolls (containing milk and eggs)	4.5x	
Mashed potatoes (containing milk and/or butter)	2.0x	
Egg noodles	2.0x	

Data adapted from Chungchunlam et al. (22) and Chungchunlam et al. (23).

The nutrients that were supplied by both baseline (animal- and plant-containing foods) and plant-only modeled least-cost dietary scenarios at exactly their minimum requirements, were the essential fatty acid α-linolenic acid, potassium, choline, vitamin D, and vitamin E. Compared to being close to limiting in the baseline least-cost diet, vitamin C and vitamin K were adequately provided by the plant-only least-cost diet. A nutrient that was supplied at its minimum required level by the plant-only least-cost diet was pantothenic acid.

#### 2.1.1 Protein quality of least-cost diets in the United States

Protein quality is considered to be a potentially important factor for assessing the inclusion levels of animal and plant food protein sources in least-cost dietary patterns. The protein quality of a food is dependent on its amino acid composition and the bioavailability of the dietary protein and dietary indispensable amino acids (27). Amino acid bioavailability in humans is best expressed as true (standardized) ileal amino acid digestibility, determined at the end of the small intestine rather than over the total digestive tract and corrected for endogenous amino acid losses (27, 28). The protein quality of least-cost diets in the US was not reported in our previous study (22). In our previous work, we described the amino acid composition of the foods on a gross, and not on a digestible basis, and it is thus relevant to explore potential effects of differences in amino acid digestibility among food types. To this end, amino acid contents of foods found in the LP modeled least-cost dietary patterns were corrected here for true ileal amino acid digestibility (29), and digestible indispensable amino acid scores (DIAAS) were calculated (27) and used to estimate the amount of utilizable protein in the least-cost diets.

The recommended dietary allowance (RDA) for protein for an average US adult, with a reference body weight of 70 kg for US adult men and 57 kg for US adult women, and a recommended protein intake of 0.80 g/kg body weight/day, is estimated to average 51 g of utilizable protein, as given in terms of bioavailable amounts of dietary protein that the adult human body can use (30). Based on the gross dietary protein and amino acid contents, the requirements for protein were sufficiently met by the baseline least-cost diet (89.4 g of gross protein, 176% of RDA) and the plant-only least-cost diet (77.2 g of gross protein, 152% of RDA). Dietary protein was mostly provided by legumes (27.6%) and milk (26.8%) in the baseline least-cost diet, and by legumes (27.8%) and soymilk (19.2%) in the plant-only least-cost diet, respectively.

**Figure 1:**
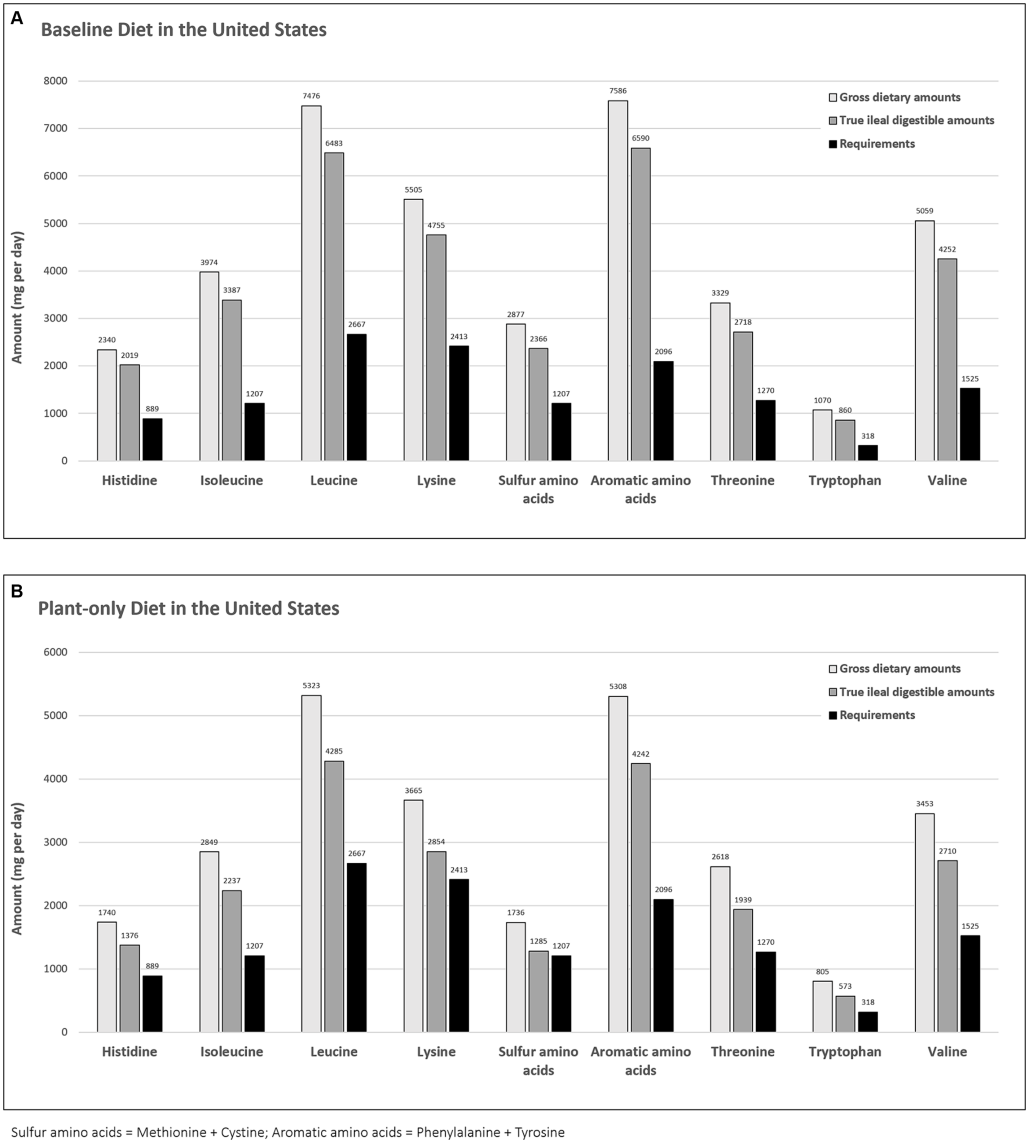
Daily indispensable amino acid requirements for an average adult (mg per day) vs. gross and true ileal digestible dietary amounts (mg per day) for the baseline **(A)** and plant-only **(B)** least-cost diets in the United States.

Similarly, and when based on the LP analysis using gross dietary protein and amino acid contents, the indispensable amino acids were well supplied by the baseline least-cost diet (228–362% of RDA) and plant-only least-cost diet (144–253% of RDA). When corrections were made independently for true ileal amino acid digestibility, the indispensable amino acids still exceeded their nutritional requirements, but by lower proportions than when expressed on a gross dietary basis (Figure 1). True ileal digestible amino acid requirements were adequately met by the consumption of the baseline least-cost diet (196–314% of RDA) and plant-only least-cost diet (106–202% of RDA). Importantly, true ileal digestible sulfur amino acids (methionine + cystine) were at only 106% of their required level, when supplied by the plant-only least-cost diet.

DIAAS is the currently recommended method for dietary protein quality assessment and for calculation, requires a reference amino acid scoring pattern for the indispensable amino acids (Table 3). The Food and Agriculture Organization (FAO) has published amino acid scoring patterns for the calculation of DIAAS (27), and comparisons were made here using the recommended reference patterns for adults aged over 18 years old (FAO adult). It is important to note, however, that DIAAS is often based for regulatory purposes, on the amino acid reference pattern of a child aged 6 months to 3 years (FAO young child) (27), and this reference pattern was also applied in the present analysis. It was estimated that the DIAAS for the baseline least-cost diet, in relation to the FAO amino acid scoring patterns for the adult and young child, was 120 and 95%, respectively (Table 3). The baseline least-cost diet provided adequate amounts of utilizable protein and indispensable amino acids, though true ileal digestible lysine was supplied at its lowest level. However, for the modeled plant-only least-cost diet, the DIAAS was 76% for the FAO adult reference pattern, and 62% for the FAO young child reference pattern (Table 3). The plant-only least-cost diet was estimated to contain respective amounts of 58.4 and 47.6 g of utilizable protein, and was potentially limiting for the sulfur amino acids in their digestible form. The current analysis highlights that protein quality is an important consideration when assessing sustainable diets, particularly for plant-sourced diets, and amino acid digestibility needs to be taken into account.

**Table 3:** Calculation of digestible indispensable amino acid score (DIAAS) values for the baseline and plant-only least-cost diets in the United States, and the recommended reference amino acid scoring patterns against which DIAAS was calculated.

		His	Ile	Leu	Lys	SAA	AAA	Thr	Trp	Val	
**True ileal digestible amino acid content of least-cost diets (mg/g protein)**											
	Baseline Diet	22.9	38.4	73.5	53.9	26.8	74.7	30.8	9.7	48.2	
	Plant-only Diet	18.0	29.3	56.1	37.4	16.8	55.6	25.4	7.5	35.5	
**Reference amino acid scoring patterns (mg/g protein)**											
	FAO adult^1^	15	30	59	45	22	38	23	6.0	39	
	FAO young child^2^	20	32	66	57	27	52	31	8.5	43	
**Digestible indispensable amino acid reference ratio** ^**3**^											**DIAAS (%)** ^**4**^
	**Baseline diet**										
	FAO adult	1.53	1.28	1.25	1.20	1.22	1.96	1.34	1.62	1.24	120
	FAO young child	1.14	1.20	1.11	0.95	0.99	1.44	0.99	1.15	1.12	95
	**Plant-only diet**										
	FAO adult	1.20	0.98	0.95	0.83	0.76	1.46	1.10	1.25	0.91	76
	FAO young child	0.90	0.92	0.85	0.66	0.62	1.07	0.82	0.88	0.83	62

His, Histidine; Ile, Isoleucine; Leu, Leucine; Lys, Lysine; SAA, Sulfur amino acids; Methionine + Cystine; AAA, Aromatic amino acids; Phenylalanine + Tyrosine; Thr, Threonine; Trp, Tryptophan; Val, Valine; DIAAS, digestible indispensable amino acid score.

1 FAO adult reference pattern is based on the amino acid scoring patterns for adults aged over 18 years old, as recommended by the Food and Agriculture Organization (FAO) (27).

2 FAO child reference pattern is based on the amino acid scoring patterns for young children aged 6 months to 3 years, as recommended by the Food and Agriculture Organization (FAO) (27). For regulatory purposes, this scoring pattern for young children is recommended for the calculation of DIAAS.

3 Digestible indispensable amino acid reference ratio is obtained from the true ileal digestible indispensable amino acid content in 1 g of dietary protein (mg/g protein) divided by the same indispensable amino acid in 1 g of reference protein (mg/g protein), for a given reference amino acid scoring pattern.

4 DIAAS, expressed as a percentage (%), is the lowest calculated digestible indispensable amino acid reference ratio multiplied by 100, for a given reference pattern.

### 2.2 Least-cost diets in New Zealand

While the LP modeling study focused only on the US (22), the US government provides economic subsidies to the animal-sourced food sector (31). This may distort the US food market and affect the relative prices of animal-sourced foods compared with the retail prices of plant-based commodity crops (32, 33), which in turn would influence the outcomes of our LP analyses. Using food prices in the New Zealand (NZ) market, another LP modeling study was conducted, where the eating habits and food economic status are similar to the US, but where food subsidies imposed on animal-sourced foods are not found.

In agreement with the US LP modeling study, foods sourced from animals, such as dairy milk, eggs, and seafood, were found in the least-cost diet in NZ. The nutrient adequate baseline least-cost diet had a daily diet cost of NZ $ 3.23 (2020 NZ food prices; US $ 2.14), and the main contributors to total diet cost among the 13 foods were legumes (29%), milk (21%), and seeds (13.0%) (23). A plant-only nutrient adequate least-cost diet, with a daily diet cost of NZ $ 4.34 (US $ 2.87) (23), was modeled after 1.05 to 10.30-times increases in the prevailing retail prices of selected animal-sourced foods (Table 2). The majority of the total diet cost contribution by the plant-based foods was from fortified soymilk (47%), seeds (12.6%), pasta (10.5%), and legumes (7.3%). The essential nutrients that were commonly first-limiting in both dietary scenarios were calcium, selenium, biotin, pantothenic acid, vitamin A, and vitamin C, with the plausible addition of potassium. While molybdenum was found to be supplied well in excess of requirements, zinc, vitamin B-12, and vitamin D were found to be first-limiting when the least-cost diet was formulated with plant-sourced foods only.

### 2.3 Least-cost diets in developing countries

The above findings are specific to high-income countries, such as the US and NZ, and may not apply to developing low- and middle-income countries (8–17, 34, 35). In developing countries, the prices of animal-sourced foods may be relatively higher than the prices of plant-sourced foods. A LP modeling study in Indonesia has shown that dairy milk, chicken liver, and clams are needed in a least-cost diet, for the adequate provision of calcium, sodium, potassium, and vitamin A, for a daily diet cost of Rp 16,189 (US $ 1.09). These results are preliminary, and should be viewed with some caution, but do mirror the results found for developed economies. Similar LP studies are currently being undertaken by our research group for the Philippines, Kenya, and Tanzania. Such countries are highly vulnerable to changes in food prices (8–11, 14, 17, 34, 35).

## 3 Discussion and conclusion

The economic dimension of sustainable diets that have a low environmental impact and provide socio-culturally acceptable and nutrient-dense foods, is often not considered. The focus of this paper was to review how the economic (monetary) cost of animal- and plant-sourced foods influences their inclusion in affordable least-cost mixed diets. Using the LP approach to identify foods included in modeled economically optimal least-cost diets that meet the nutrient requirements of a healthy average adult, animal-sourced foods were selected under current market conditions in the US and NZ. Foods originating from animals, such as dairy milk, eggs, fish, and seafood, were often key components of the least-cost diets. Legumes, milk, potatoes, and seeds were the greatest contributors to diet cost, whereas fats, oils, sugars, and starchy staples were low-cost rich sources of energy. As these findings are relevant to developed high-income countries, as exemplified by the US and NZ, there is an urgent need for LP modeling studies to test the premise that animal-sourced foods will be included in such least-cost diets in developing countries. Preliminary modeling studies in Indonesia indicate that animal-sourced foods, such as dairy milk, chicken liver, and seafood, are required for the least-cost diet, and the same LP modeling approach is currently being applied in the Philippines, Kenya, and Tanzania.

Concomitantly, in these studies, a number of dietary scenarios were analyzed that involved relaxing food price constraints around the foods included. The magnitude of food price elasticities by which the prices of animal-sourced foods needed to rise to be excluded from least-cost dietary patterns was estimated to formulate an explorative scenario of a plant-only least-cost dietary pattern. In the US, the prevailing retail prices of all animal-based foods had to be increased by 2.0 to 11.5 times their baseline costs to generate a plant-only least-cost diet, that had a diet cost that was 45% higher than that for the least-cost diet that contained animal- and plant-derived foods. Similar results were found for NZ, where the market prices of animal-based foods are not subjected to government subsidies to the same extent as in the US (31–33). When the baseline prices of animal-sourced foods were increased by 1.05 to 10.30 times, a least-cost diet with only plant-based foods was modeled, with a daily diet cost that was 34% more than that of the least-cost diet that contained animal- and plant-sourced foods. These results, representative of the US and NZ markets, give a clear indication of the leeway of food price variations of these animal foods for their complete exclusion from least-cost dietary patterns. Such food retail price interventions in developing low- and middle-income countries merits more investigation. As diet costs were limited to average annual national retail food prices, more in-depth country-level food prices are needed to consider regional diversity, seasonal and monthly variations, and affordability differences at a household level. In addition, while the cost of diets in this case relates to the market cost of food to the consumer, externality food costs include cost associated with food production, food processing and transportation, and food waste. Trade-offs may be appropriate to potentially cover these wider food costs.

The foods selected in the LP modeled dietary patterns are not meant to be necessarily included in realistic diets for consumption, but merely were identified to fulfill the arbitrary requirements for energy and country-specific dietary nutrient recommendations for almost all individuals in an average adult population aged 19–50 years (22, 23). Further modeling research for the elderly, pregnant or lactating women, adolescents, and growing children, who are most susceptible to inadequate nutrient intakes and increases in food prices (9–11, 16–18, 36, 37) warrants investigation. The first-limiting nutrients were found to be mostly the vitamins and minerals, notably calcium, potassium, selenium, vitamin A, vitamin C, vitamin D, and vitamin E. Particular additional nutrients that were first-limiting in the plant-only dietary scenarios were zinc, pantothenic acid, and vitamin B-12 (22, 23, 38, 39). It is of considerable note that most plant-sourced foods in the modeled least-cost diets were enriched with essential vitamins and minerals. For instance, fortified soymilk was the predominant source of plant-based vitamin B-12 in NZ (23). Fortification of plant-sourced foods has secured their place in the modeling of plant-based nutrient adequate least-cost dietary patterns.

Nutritional adequacy depends on the dietary supply of nutrients and bioavailability, that can be described as the proportion of an ingested nutrient that is available for utilization in metabolic functions (39). Natural food products originating from animals often contain protein and key vitamins and minerals, in higher amounts and greater bioavailability, than those of plant origin (27, 29, 39–44). Regarding protein quality, in general, least-cost diets that included animal proteins scored higher on DIAAS than least-cost diets that contained only plant-based protein sources. When all animal proteins were replaced with plant proteins in the US, utilizable protein intake was greatly reduced. Consideration of such protein quality metrics suggests that animal proteins play a critical role for ensuring sufficient provision of utilizable protein and indispensable amino acids (45, 46). The question also remains as to whether incorporating bioavailability of vitamins and minerals, that varies greatly among animal and plant food sources, will significantly impact the composition and cost of nutritionally adequate least-cost dietary patterns. The outcomes and conclusions may substantially change when diet cost is expressed per g of nutrient, and more importantly per g of bioavailable nutrient (46–48), in the LP modeling studies.

Taking an economic sustainability perspective toward dietary patterns in the US and NZ, and preliminarily in Indonesia, animal-sourced foods needed to be included in least-cost diets, to sufficiently meet basic nutrient requirements of the adult population, at the lowest retail dietary cost. Our results show that animal-derived foods are economically valuable sources of first-limiting essential key vitamins and minerals, and there is a considerable margin whereby the prevailing prices of animal-sourced foods need to increase to ensure their exclusion. Furthermore, when all animal-based foods were substituted with plant-based foods, the modeling of exclusively plant-sourced nutrient adequate least-cost dietary patterns was dependent on nutrient fortification and was relatively expensive. The respective roles of animal and plant food sources for the affordable and adequate provision of essential nutrients, and the often-missing economic dimension in the context of sustainable nutrition security, has been addressed.
